# Molecular characterization of lung squamous cell carcinoma tumors reveals therapeutically relevant alterations

**DOI:** 10.18632/oncotarget.27905

**Published:** 2021-03-16

**Authors:** Asim Joshi, Rohit Mishra, Sanket Desai, Pratik Chandrani, Hitesh Kore, Roma Sunder, Supriya Hait, Prajish Iyer, Vaishakhi Trivedi, Anuradha Choughule, Vanita Noronha, Amit Joshi, Vijay Patil, Nandini Menon, Rajiv Kumar, Kumar Prabhash, Amit Dutt

**Affiliations:** ^1^Integrated Cancer Genomics Laboratory, Advanced Centre for Treatment Research Education in Cancer (ACTREC), Tata Memorial Centre, Navi Mumbai, Maharashtra 410210, India; ^2^Department of Medical Oncology, Tata Memorial Centre, Ernest Borges Marg, Parel, Mumbai, Maharashtra 400012, India; ^3^Department of Pathology, Tata Memorial Centre, Ernest Borges Marg, Parel, Mumbai, Maharashtra 400012, India; ^4^Homi Bhabha National Institute, Training School Complex, Anushakti Nagar, Mumbai, Maharashtra 410210, India; ^5^Centre for Computational Biology, Bioinformatics and Crosstalk Laboratory, ACTREC, Tata Memorial Centre, Navi Mumbai, Maharashtra 410210, India

**Keywords:** lung squamous carcinoma, genetic alterations, druggable mutations, whole exome sequencing, mass spectrometry

## Abstract

Introduction: Unlike lung adenocarcinoma patients, there is no FDA-approved targeted-therapy likely to benefit lung squamous cell carcinoma patients.

Materials and Methods: We performed survival analyses of lung squamous cell carcinoma patients harboring therapeutically relevant alterations identified by whole exome sequencing and mass spectrometry-based validation across 430 lung squamous tumors.

Results: We report a mean of 11.6 mutations/Mb with a characteristic smoking signature along with mutations in *TP53 (65%), CDKN2A (20%), NFE2L2 (20%), FAT1 (15%), KMT2C (15%), LRP1B (15%), FGFR1 (14%), PTEN (10%)* and *PREX2* *(5%)* among lung squamous cell carcinoma patients of Indian descent. In addition, therapeutically relevant *EGFR* mutations occur in 5.8% patients, significantly higher than as reported among Caucasians. In overall, our data suggests 13.5% lung squamous patients harboring druggable mutations have lower median overall survival, and 19% patients with a mutation in at least one gene, known to be associated with cancer, result in significantly shorter median overall survival compared to those without mutations.

Conclusions: We present the first comprehensive landscape of genetic alterations underlying Indian lung squamous cell carcinoma patients and identify *EGFR, PIK3CA, KRAS* and *FGFR1* as potentially important therapeutic and prognostic target.

## INTRODUCTION

Lung cancer is the leading cause of cancer-related deaths across the globe with more than 1.7 million deaths annually [1]. In India, lung cancer contributes to 8.1% of all cancer-related deaths [1]. Non-small cell lung cancer (NSCLC), more common type of lung cancer, accounts for 85% of all lung cancers comprise of two major histological subtypes, adenocarcinoma and squamous cell carcinoma [2]. The adenocarcinoma of the lung arises mostly in patients with no previous significant tobacco exposure, while the squamous subtype is found almost exclusively in former or current smokers [3] with relatively higher overall mutational load [4]. Despite distinct histological and biological characteristics, the two NSCLC subtypes are largely treated with the same chemotherapeutic agents except for pemetrexed to treat non-squamous NSCLC [5]. Significant advances in the molecularly targeted therapies have been made to treat lung adenocarcinoma patients harboring mutant *EGFR*, *ALK, RET,* *ROS1*, *BRAF*, *MET*, *NTRK-1 & 2*, *ERBB2*, and *FGFR*3 [6–8]. However, no approved targeted therapy regimens are available for lung squamous patients in spite of distinct genetic alterations identified in the tumor type, including alterations in *TP53, PIK3CA, CDKN2A, MLL2, PTEN,* *KEAP1, NFE2L2,DDR2,* *FGFR1, PDGFRA, SOX2*, and *CCND1* [9–15]. Moreover, most of the reported studies describe Caucasian, Chinese, Korean and Japanese population, with sparse information on the molecular profile of lung squamous patients of Indian origin that accounts for about 30% of Indian lung cancer disease [16].

In this study, we sought to describe the first genetic landscape of alterations underlying 430 Indian lung squamous genomes and uncover the prevalence of known targetable somatic alterations using next generation sequencing followed by validation using mass spectrometry.

## RESULTS

### Genomic landscape of alterations in lung squamous carcinoma samples

**Figure 1:**
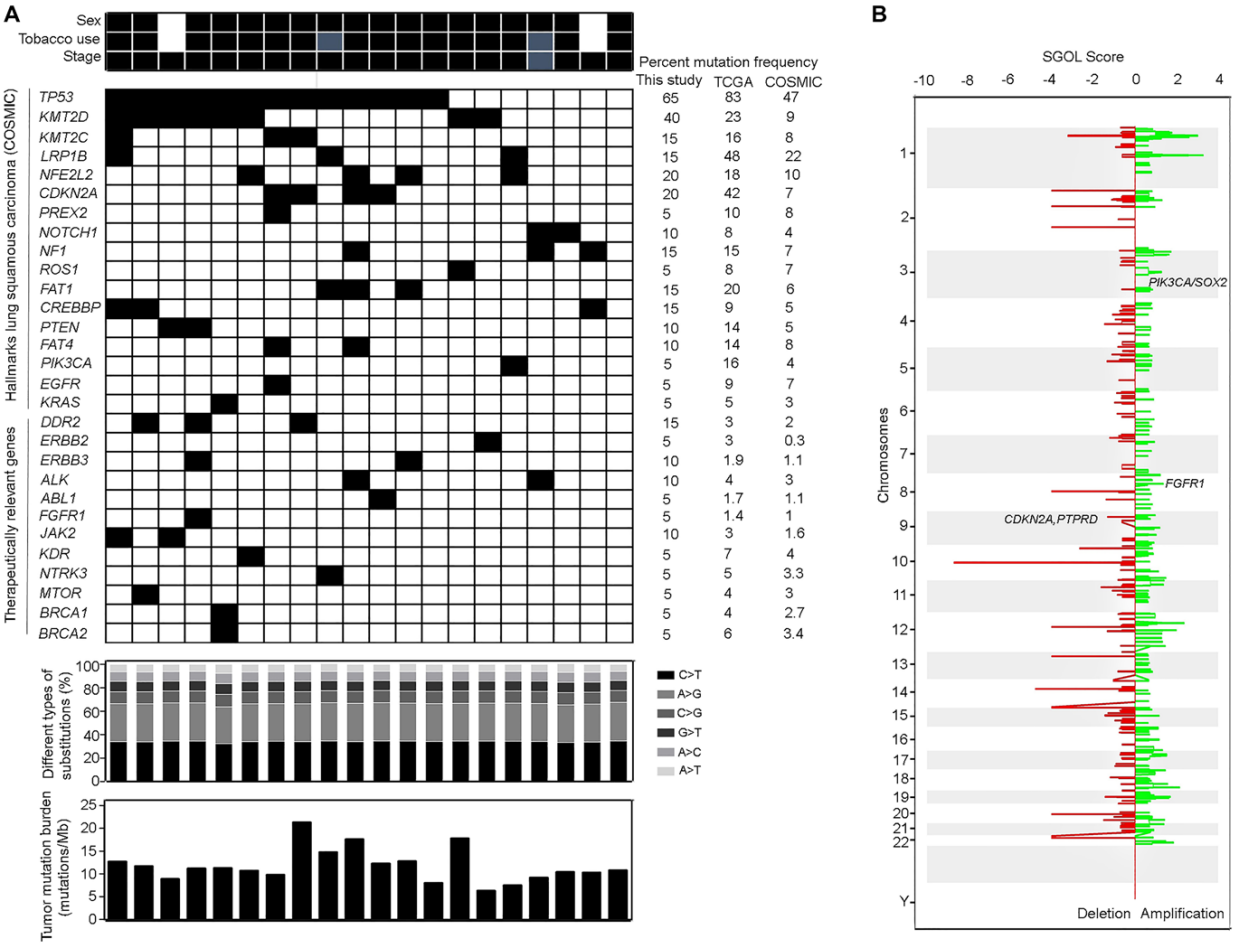
Somatic mutations and copy number variations observed in lung squamous carcinoma patients. (**A**) The spectrum of mutations obtained after whole exome sequencing analysis of discovery set of 20 Indian origin lung squamous carcinoma patients is represented in the form of heatmap. Black solid box indicates the patient samples positive for the mutation in the specified genes and white box indicates the wildtype status of the particular gene. The clinical characteristics of the patients including the sex, tobacco use and stage are mentioned above the heatmap. Males, tobacco users and stage IV tumors are indicated by black solid boxes while females, non-tobacco users are indicated by white boxes. Grey boxes indicate information not available. Alterations in genes known to be hallmarks of lung squamous carcinoma (based on COSMIC) alongwith therapeutically relevant genes observed in our dataset are depicted. The mutation frequencies of the genes observed in this study (*n* = 20) are compared with those already reported in COSMIC (*n* > 1000) and TCGA (*n* = 587) databases. Additional characteristics of the whole exome data including the distribution of different types of transitions and transversions (according to different shades as specified in the color code) and the tumor mutation burden (mutations/Mb) are shown in the bar graphs below the heatmap. (**B**) Somatic copy number changes obtained from whole exome sequencing data based on CODEX2 pipeline. The score for segment gain or loss (horizontal axis) is plotted for each chromosome (vertical axis) and represents copy number gain (positive SGOL score, green color) or loss (negative SGOL score, red color). The representative hallmark cancer associated genes are mentioned in the respective amplified/deleted regions.

We performed whole exome sequencing of 20 lung squamous tumors – pathologically confirmed to have tumor content above 40%, at an average on-target coverage of 50–70X, followed by mass spectrometry-based genotyping of 430 tumors (Table 1 and Supplementary Table 1) using a customized panel of 53 hotspot mutations across 17 genes (Supplementary Table 2). The sequencing quality, tumor purity and characteristic features of exome analysis for all the samples are detailed in Supplementary Tables 3 and 4. The exome analysis revealed a non-synonymous mean somatic mutation rate of 11.6 mutations/Mb and median of 11 mutations/Mb with enrichment of C>T transitions as opposed to putative C>A transversions indicative of a smoking signature [17, 18] . However, these C>T transitions did not show concordance (cosine similarity > 0.9) with any of the 30 signatures defined in COSMIC. A total of 9261 alterations including 4181 missense mutations, 3658 indels, 837 splice-site mutations and 585 nonsense and nonstop alterations were observed. Consistent with the literature, *TP53* was identified to be the most commonly mutated gene (Figure 1) at a frequency of 65%. Similarly, mutations in *CDKN2A* (20%) and *PTEN* (10%) were observed with co-occurring *TP53* alteration, as reported earlier [9, 12, 15]. Additionally, alterations in tumor suppressor genes including *NFE2L2* (20%, 4 cases), *KMT2C* (15%, 3 cases), *LRP1B* (15%, 3 cases), *FAT1* (15%, 3 cases), *NF1* (15%, 3 cases) and *PREX2* (5%, 1 case) were observed at comparable frequencies, as reported earlier [9, 11] (Figure 1). Interestingly, alterations in *KMT2D* were altered at a higher frequency of 40%. The complete list of the somatic substitutions obtained from exome analysis is detailed in Supplementary Table 5.

**Table 1:** Demographic characteristics of lung squamous patients

Total number of lung squamous patients	430
**Age**	
< 65 years	283
> 65 years	147
Mean	59.5 (21–85 years)
**Gender**	
Male	364 (84%)
Female	66 (16%)
**Habits**	
Smokers	309 (72%)
Non-smokers	53 (12%)
Not available	68
**Stage**	
Stage IV	208 (48%)
Stage III	83 (19%)
Stage II	25 (6%)
NA	114

Inferred copy number analysis based on the exome data identified previously described copy number amplifications harboring genes including *FGFR1* (10%), SOX2/ *PIK3CA* (5%), *MYC* (10%), *CDK6* (10%) and *MET* (5%) (Figure 1B) [9, 17, 19, 20]. *FGFR1* amplification is therapeutically relevant hallmark alteration in lung squamous cancer [20]. Thus, based on the availability and quality of genomic DNA samples, we selected a subset of 100 patients to validate copy number status at *FGFR1* amplification in these patients using real-time PCR. We observed *FGFR1* amplified in 14% of Indian Lung squamous patients (Supplementary Figure 1).

**Figure 2:**
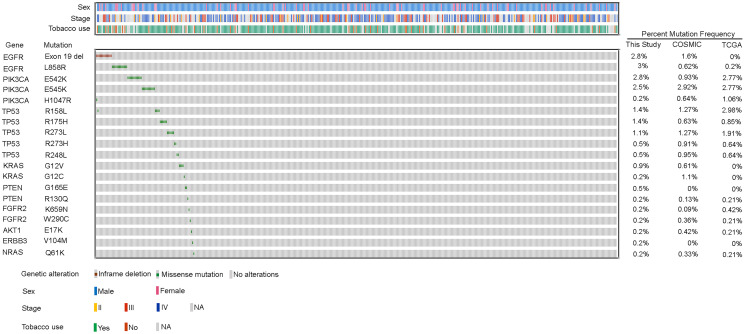
Recurrent genetic alterations in Indian lung squamous carcinoma patients. Heatmap representation of genetic alterations identified by mass spectrometry based genotyping in 430 lung squamous patients. The clinical characteristics of the patients including the sex, tobacco use and stage are mentioned above the heatmap and the respective color codes are mentioned below the heatmap. Only alterations observed at a frequency > 0 are depicted in the heatmap. Alterations which were a part of panel, but not observed in any of the samples are not included. Comparison of frequencies of each alteration between our study and those reported in COSMIC and TCGA databases is shown on the right side of the heatmap.

Similarly, activating kinase domain mutations of *EGFR*, including the in-frame 15bp deletion in exon 19, *EGFR* E746_A750 del and point mutation in exon 21, *EGFR* L858R were observed at a cumulative frequency of 5.8% (Figure 2) across 25/430 tumors (Supplementary Table 6). Interestingly, the activating mutations in the exon 18 of *EGFR* were not observed in any of the tumors, as reported earlier [9, 14]. In overall, *EGFR* activating mutations (5.8%) were observed at a significantly higher frequency than the Caucasians (0.2%, *p* = 0.0005, *n* = 487) [9] , Korean population (0.1%, *p* = 0.0405, *n* = 104) [12] and Chinese populations (3.7%, *p* = 0.285, *n* = 271) [11, 21]. The activating *KRAS* mutations affecting the codon 12, observed at a frequency of 1.1% in our cohort, were mutually exclusive to *EGFR* mutations (*p* = 0.37, E*GFR* = 25, *KRAS* = 5, both *EGFR* and *KRAS* = 0, neither = 400). The canonical *PIK3CA* mutations, characteristic to lung squamous tumors, were observed at a frequency of 5.5% (E542K: 2.8%, E545K: 2.5% and H1047R: 0.2%, Supplementary Table 6), comparable to as reported among the Caucasian population [9, 14]. Interestingly, of the 49 samples harboring either *PIK3CA* or *EGFR* alterations, only one sample showed co-occurring *EGFR* L858R and *PIK3CA* H1047R mutation. However, the mutual exclusivity of mutations among these two genes was not found to be significant (*p* = 0.575, E*GFR* = 25, *PIK3CA* = 23, both *EGFR* and *PIK3CA* = 1 neither 381). Furthermore, activating mutations at a lower frequency were observed in other genes including *FGFR2* (0.5%), *AKT1* (0.2%), *NRAS* (0.2%) and *ERBB3* (0.2%) (Supplementary Table 6). Somatic mutations probed in other oncogenes *CTNNB1, DDR2, ERBB2* and *FGFR3* were not observed in any of the 430 tumor samples.

### Clinical correlation with genetic alterations in lung squamous cancer

**Figure 3:**
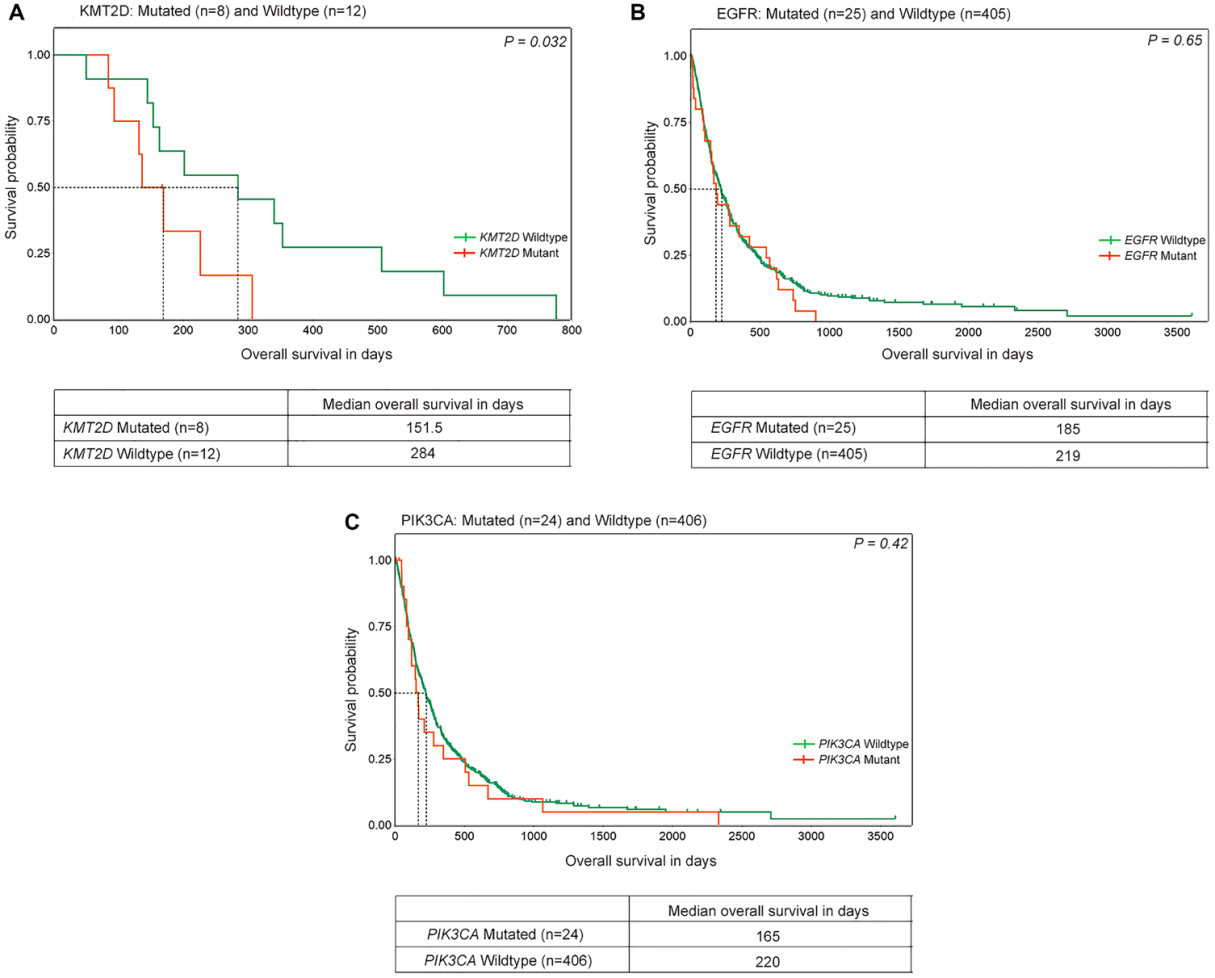
Overall survival in lung squamous tumors with distinct gene alterations. Kaplan-Meier plots of overall survival (in days) in lung squamous tumors with mutations in (**A**) KMT2D (**B**) EGFR and (**C**) PIK3CA are shown. The orange and green lines denote mutated and wild-type patients respectively. The dotted lines indicate median overall survival of the respective groups. *P-value* is indicated on the top right corner of the plots. The number of patients and median survival in each group is mentioned in the table below.

**Figure 4:**
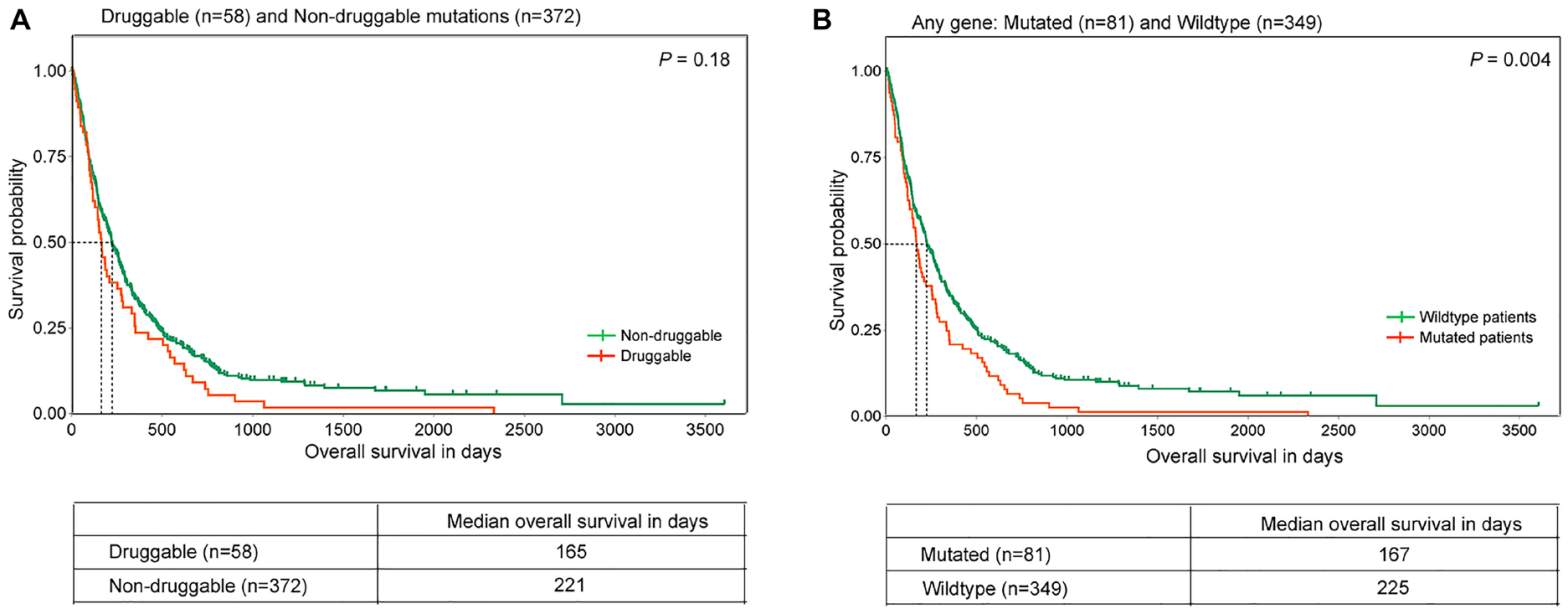
Overall survival in lung squamous tumors with distinct gene alterations. Kaplan-Meier plots of overall survival (in days) in lung squamous tumors with mutations in (**A**) Druggable genes and (**B**) any gene are shown. The orange and green lines denote mutated and wild-type patients respectively. The dotted lines indicate median overall survival of the respective groups. *P-value* is indicated on the top right corner of the plots. The number of patients and median survival in each group is mentioned in the table below.

Our study did not reveal any significant association between clinical features such as age, gender, tumor stage, smoking, tobacco usages with mutations across any of the 17 genes probed in the genotyping experiment, other than fourfold higher *EGFR* mutations (*p* = 0.001), and twofold higher mutations (*p* = 0.02) among non-smokers (Table 2 and Supplementary Table 7), consistent with literature [21]. Next, we analysed whether these mutations are associated with the disease prognosis. Of all the mutations analyzed, the median overall survival of *KMT2D* mutated patients was found to be significantly lower than among patients with *KMT2D* wildtype (151.5 days, 284 days, *p* = 0.032) (Figure 3A), as reported earlier [11, 12]. Similarly, *EGFR* and *PIK3CA* mutations were associated with poor prognosis with a median overall survival of 185 and 165 days compared to 219 and 220 days for patients with wildtype, respectively (Figure 3B and 3C), irrespective of their smoking status. Of note, the association of *EGFR* and *PIK3CA* mutations and the lower overall survival of lung squamous carcinoma patients however did not attain statistical significance, likely due to lower incidence of the alterations. Additionally, 13.5% of lung squamous patients harboring druggable oncogenic mutations (including *KRAS G12C*) showed lower median overall survival (165 days) compared to patients harboring other mutations, (221 days) (Figure 4A). And, of most significance, 19% patients harboring a mutation in at least one gene, known to be associated with lung squamous cancer, as inferred by mass-spectrometry based genotyping resulted in significantly shorter median overall survival compared to lung squamous patients with no mutations (167 days vs 225 days, Figure 4B), wherein no patient received any targeted therapy.

**Table 2:** Corelation of clinicopathological features and gene alterations in Indian lung squamous patients

Clinico- pathological features	Variable	Number (%), along the column	Gene alterations from mass-array based genotyping, Number (%) along the row														
			EGFR		*P* value	PIK3CA		*P* value	Oncogenic mutations		*P* value	Any mutation of panel		*P* value
			Mutant	Wildtype		Mutant	Wildtype		Mutant	Wildtype		Mutant	Wildtype	
Age	< 65 years	283	21 (7.4%)	262 (92.6%)	0.051	16 (5.6%)	267 (94.4%)	1	43 (15%)	240 (85%)	0.18	59 (21%)	224 (79%)	0.153
	> 65 years	147	4 (2.7%)	143 (97.2%)		8 (5.4%)	139 (94.6%)		15 (10%)	132 (90%)		22 (15%)	125 (85%)	
Gender	Females	66	7 (10.6%)	59 (89.4%)	0.084	6 (9%)	60 (91%)	0.236	13 (19.6%)	53 (80.4%)	0.118	14 (21%)	52 (79%)	0.608
	Males	364	18 (5%)	346 (95%)		18 (5%)	346 (95%)		45 (12.3%)	319 (87.7%)		67 (18.4%)	297 (81.6%)	
Tumor stage	II	25	1 (4%)	24 (96%)	0.828	2 (8%)	23 (92%)	0.32	4 (16%)	21 (84%)	0.651	6 (24%)	19 (76%)	0.373
	III	83	5 (6%)	78 (94%)		2 (2.4%)	81 (97.6%)		8 (9.6%)	75 (90.4%)		13 (15.6%)	70 (84.4%)	
	IV	208	9 (4.3%)	199 (95.7%)		12 (5.7%)	196 (94.3%)		23 (11%)	185 (89%)		30 (14.4%)	178 (85.6%)	
	Information not available	114	10	104		8	106		23	91		32	82	
Smoking status	Non-smoker	53	8 (15%)	45 (85%)	**0.002**	4 (7.5%)	49 (92.5%)	0.512	12 (22.6%)	41 (77.4%)	**0.022**	16 (30%)	37 (70%)	**0.022**
	Smoker	309	11 (3.5%)	298 (96.5%)		16 (5%)	293 (95%)		33 (10.6%)	276 (89.4%)		51 (16.5%)	258 (83.5%)	
	Information not available	68	6	62		4	64		13	55		14	54	

## DISCUSSION

We earlier reported a distinct frequency of 23% *EGFR* mutations and 18% *KRAS* mutations in lung adenocarcinoma patients of Indian ethnicity compared to 10–15% vs. 27–62% *EGFR* mutations and 25–50% vs. 5–15% *KRAS* mutations among the Caucasians and East Asians populations [6, 22–24]. These studies underscore the somatic mutation variability in lung cancer across populations. Large scale genomic analyses have identified alterations that underlie squamous cell lung cancers [10, 11, 25]. However, most of the lung squamous studies described so far includes mainly the Caucasian, Chinese, Korean and Japanese population, while the molecular profiling of lung squamous patients of Indian origin remains sparsely explored. Here, we describe the first comprehensive landscape of genomic alterations prevalent in Indian lung squamous patients using a combination of next generation sequencing and genotyping technologies. Our study is marked by a deficiency of a lower average on-target coverage of 50–70X of orphan FFPE tumors. However, several lines of distinct features underlie this study attributing to unique etiology and specific population, which has been previously described for early stage tongue tumors among patients of Indian origin [26].

To begin with, we observed a relatively higher non-synonymous mean somatic mutation rate of 11.6 mutations/Mb compared to 8.1 mutations/ Mb among Caucasian population, 8.7 mutations/Mb among Korean population and 7.1 mutations/Mb among Chinese population, which is also considerably higher than the mutation rate observed in various non-tobacco associated cancer types [9, 12]. Interestingly, our data suggests an enrichment of C>T transitions as opposed to putative C>A transversions indicative of a smoking signature [17, 18] consistent with our previous report in tongue squamous tumors [26] and gingiva-buccal tumors as reported by the ICGC-India [27] with tobacco chewing habit. Furthermore, our lung squamous data is largely consistent with the “squamousness” characteristic alterations as described underlying all squamous tumors arising across different anatomical sites [28] — such as *TP53*, *PIK3CA*, *CDKN2A*, and *SOX2*. However, we observed a significant exception with the absence of alterations at the *CCND1* loci among the squamous cell lung carcinoma patients from India compared to 7% frequency among Caucasian patients [29]. Of other significant alterations known to occur in lung squamous, we observed 65% *TP53* alterations in our study as reported across different ethnic groups [9–14]. Also, alterations in other tumor suppressor genes, including *CDKN2A (20%), NFE2L2 (20%), FAT1 (15%), KMT2C (15%)*, *LRP1B (15%)*, *PTEN* (10%) and *PREX2 (5%)* were comparable to earlier reports [9–12, 14, 17].

Among the therapeutically relevant alterations, frequent oncogenic alterations were found in the PI3K-AKT pathway at a cumulative frequency of 10.7%, as reported in other studies [9, 14]: including 5% amplification at *SOX2*/*PIK3CA*; 5.5% *PIK3CA* mutations; and, *AKT1 E17K* mutation. Interestingly, the BASALT-1 phase II trial emphasised the prognostic impact of the PI3K pathway in lung squamous cancer, suggesting *PIK3CA* alterations in lung squamous as a good prognostic marker [30]. Secondly, we found 14% lung squamous cancer patients harboring *FGFR1* amplification, comparable to as reported in other population as a promising therapeutic target [19, 20]. While a correlation between the *FGFR1* copy number and protein expression remains to be established, this study underlines the relevance of the clinical trials testing the fibroblast growth factor receptor inhibitors in clinical use for the treatment of lung squamous cancer [31, 32]. Despite the dismal survival benefit of the LUME-Lung 1 trial in unselected advanced lung squamous cancer patients trial with combination of FGFR inhibitor with chemotherapeutic agent, the findings of this study along with preclinical studies [33, 34], suggest that pre-selection of ~14% lung squamous patients harboring *FGFR1* alterations are more likely to benefit from the treatment; Thirdly, significant alterations in *KMT2D* were observed at a frequency of 40% compared to 10% and 24%, compared to the Caucasian and East Asian population [12, 17],based on our whole exome data that necessitate validation in a larger cohort of squamous carcinoma samples. And, lastly but most significantly, *EGFR*-tyrosine kinase inhibitor sensitive alterations were observed at a frequency of 5.8% in our cohort, significantly higher than as reported in the TCGA and studies from the East Asian populations [9–12]. As the LUX-Lung 8 trial underline benefit from the anti-*EGFR* tyrosine kinase inhibitor among lung squamous patients associated with *ERBB* alterations, the occurrence of 5.8% *EGFR* mutations among Indian lung squamous cell carcinoma patients emphasizes the potential significance and relevance of the outcome of this trial in the Indian context [35]. Taken together, 13.5% of lung squamous tumors harbored one or more mutually exclusive therapeutically relevant oncogenic mutations (including *KRAS* G12C). This is consistent with earlier reports suggesting a 10–11% frequency of potentially targetable alterations in lung squamous carcinoma [36, 37].

Our survival analysis demonstrate that lung squamous patients harboring *EGFR* or *PIK3CA* mutations have a shorter median overall survival compared to patients with no mutations; 13.5% patients harboring potentially actionable oncogenic mutations similarly have a lower median overall survival (165 days) compared to patients harboring other mutations (221 days), as reported earlier [36]; and, 19% lung squamous patients harboring a mutation in at least one gene resulted in statistically significant shorter median overall survival compared to lung squamous patients with (167 days vs 225 days). These mutations could thus help inform designing a panel of specific and actionable mutations to select patients likely to benefit from personalized treatment and follow up diagnosis based on liquid biopsy for disease progression and recurrence as shown for lung adenocarcinoma [38].

In overall, we present a striking variation of genetic heterogeneity among lung squamous cell carcinoma patients of Indian descent. The findings from this study extend the scope of the ongoing umbrella clinical trials such as the Lung-MAP master protocol that aims to evaluate multiple targeted therapeutic strategies in lung squamous cell carcinoma patients and the AACR Project GENIE database collaborative project [29, 39]. A systematic exploration of these target genes in lung squamous cell carcinoma patients and variability across ethnicity could further extend our insights into the etiology of lung squamous cancer.

## MATERIALS AND METHODS

### Patient cohort

Tumors from 430 lung squamous patients, registered at Tata Memorial Hospital, Mumbai, India during the year 2011–2016 were obtained retrospectively from the tumor tissue repository. All the tumor tissues were stored as formalin fixed paraffin embedded (FFPE) blocks as per the standard protocol of Tata Memorial Hospital, Mumbai, India. The sample cohort and the study protocols were approved by Institutional Review Board and Ethics Committee of Tata Memorial Centre-ACTREC.

### Patient details and sample information

The 430 lung squamous patient cohort comprise of 84% males and 16% females with a mean age of diagnosis at 59.5 years (range 21–84 years), 71% cases with a history of tobacco use (including former/current tobacco chewers and smokers) and tumors with stages IV (42%), III (18%) and II (7%). The medical and histopathological records including immunohistochemical staining status of p63, CK7, p40, TTF1 and Napsin A of all the patients were reviewed by oncopathologists to ensure the diagnosis of lung squamous carcinoma. Tumors diagnosed as adenosquamous tumors based on TTF1 expression were excluded from this study. The adequacy of tumor content in all the tissues was assessed by pathologists and was also inferred from whole exome sequencing dataset. All the samples had a minimum of 40% malignant cells. The complete clinical and histopathological characteristics of all the patients including age, sex, tumor stage, smoking status/tobacco usage, nature of biopsy material and immunohistochemical staining status of various markers are detailed in Table 1 and Supplementary Table 1 respectively.

### DNA extraction

Genomic DNA extraction from FFPE tumor blocks was performed according to the standard protocol of QIAmp DNA FFPE Tissue Kit. For whole exome sequencing, DNA concentration and quality was assessed using Qubit 3.0 fluorometer and Tape Station respectively and for MassArray genotyping, the DNA concentration was measured using Nanodrop 2000c spectrophotometer (Thermo Fischer Scientific Inc).

### Whole exome sequencing

Whole exome sequencing was performed on lung squamous carcinoma DNA samples by Genotypic Technology Pvt Ltd, Bengaluru, India. A minimum of 100ng input DNA (based on Qubit quantification) was utilized for library preparation. Exome capture was performed using the SureSelect Human All Exon V6 (target size 60Mb). Library preparation and exome capture was performed following the manufacturer’s instructions. Exome sequencing was performed on Illumina platform according to standard protocol using 150bp paired end reads to obtain an average coverage of 100X across all the samples.

### Exome sequencing analysis for identification of somatic mutations

The raw data was analyzed using the in-house developed pipeline as described earlier [6, 26, 40]. Variants called by the tumor only mode of both GATK and Mutect2 pipelines were combined for further analysis. As described earlier [6, 41], FFPE artefact correction was applied by removing C>T and G>A variants occurring at an allele fraction of < 5%. GATK-Funcotator (https://gatk.broadinstitute.org/hc/en-us/articles/360046786432-Funcotator) was used to annotate cancer associated variants based on which the analysis was restricted only to variants in the coding region. Germline variants were depleted based on databases including dbSNP (dbSNP_151) [42], ExAC (v0.3.1) [43], TMC-SNPdb [44], gnomAD (gnomAD v2.1.1) [45] and Genome Asia 100K while the variants either present in COSMIC (v90) [46] or none of the four databases (the novel variants) were retained. Lastly, the deleterious nature of these variants was determined using functional prediction tool based analysis on somatic non-synonymous variants using seven different tools which are part of dbNSFP v4.0a [47]. Variants called deleterious by at least four out of seven tools were considered for further analysis. The total number of non-synonymous somatic mutations within the coding region were extracted for calculation of tumor mutation burden across each tumor. The percentage of tumor cells in all the samples were computed from the exome sequencing data using the tool AITAC [48] (https://github.com/BDanalysis/aitac). Signature analysis on the exome sequencing data was performed using the R/Bioconductor package MutationalPattens [49] (http://bioconductor.org/packages/MutationalPatterns).

### Copy number analysis from exome data

Copy number variations in the whole exome data were assessed using CODEX2, a normalization based CNV detection method which works with or without matched normal samples [50]. We employed fractional mode of CODEX2 for segmentation of our data and categorized an event to be a high gain (copy number > 3.3), gain (copy number 2.3–3.3), diploid (copy number 1.7–2.3), one copy-deletion (copy number 0.7–1.7) and homozygous deletion (copy number < 0.7).

### Mass spectrometry-based genotyping

A custom panel of 53 frequently occurring hallmark mutations across 17 cancer associated genes were designed. 200ng input DNA at a final concentration of 20ng/ul from 430 Lung squamous samples was submitted to Imperial Life Sciences Pvt Ltd, New Delhi, India for validation by Mass spectrometry-based genotyping using the iPlex Pro chemistry. Using the assay design software, these 53 mutations were pooled into four wells. Accordingly, the PCR amplification and single base pair extension primers for iPLEX reaction were designed as per manufacturer’s instructions. The mutation calls were analyzed using the Typer 4.0 software and the spectra were also manually revived.

### *FGFR1* amplification using quantitative real-time PCR

Real-time primers designed to specifically amplify *FGFR1* and *GAPDH* from genomic DNA were used for real time PCR. The specificity of primers was tested by using melt cure analysis during real time PCR. The real time PCR was performed using 20 ng of genomic DNA per 6ul of reaction volume on Light cycler 480 (Roche, Mannheim, Germany). The *FGFR1* amplification for the tumor samples was calculated by normalizing the Ct values to the Ct values housekeeping control *GAPDH* and samples with normalized Ct < 1 were considered to harbor FGFR1 amplification.

### Availability of data

The FastQ files containing the raw sequence data for all the samples have been uploaded on ArrayExpress (http://www.ebi.ac.uk/arrayexpress/) hosted by the European Bioinformatics Institute under the accession number E-MTAB-8801 titled ‘Whole exome sequencing of Lung Squamous Carcinoma Patients of Indian Origin’.

### Statistical analysis

For mutation mutual exclusivity analysis, the mutant and wildtype status were defined based on the genotyping experiment and the statistical significance of the exclusivity was computed using CoMET method [51]. For correlation of clinico-pathological features and gene alterations and comparisons of mutational frequencies between different ethnic groups, Fisher exact test was used to calculate significance. In both the analysis, *P value* < 0.05 was considered significant. The Kaplan-Meier survival data analysis and clinicopathological correlation analysis was performed in R as described previously [6, 40] using R survival packages (http://cran.r-project.org/package=survival). The end point was taken as date of death wherever available, else the date of last contact was used for censoring. Tumors with mutations in genes including *EGFR, PIK3CA, KRAS, FGFR2, AKT1, NRAS and ERBB3* were grouped as therapeutically relevant alterations.

## SUPPLEMENTARY MATERIALS












