# Supplementary material for: Molecular characterization of lung squamous cell carcinoma tumors reveals therapeutically relevant alterations
Source: Oncotarget. 2021 Mar 16;12(6):578–88. doi: 10.18632/oncotarget.27905 (PMC7984830; doi:10.18632/oncotarget.27905)
Supplement: Supplementary file 1 [file oncotarget-12-578-s001.pdf]

Molecular characterization of lung squamous cell carcinoma tumors reveals therapeutically relevant alterations

SUPPLEMENTARY MATERIALS

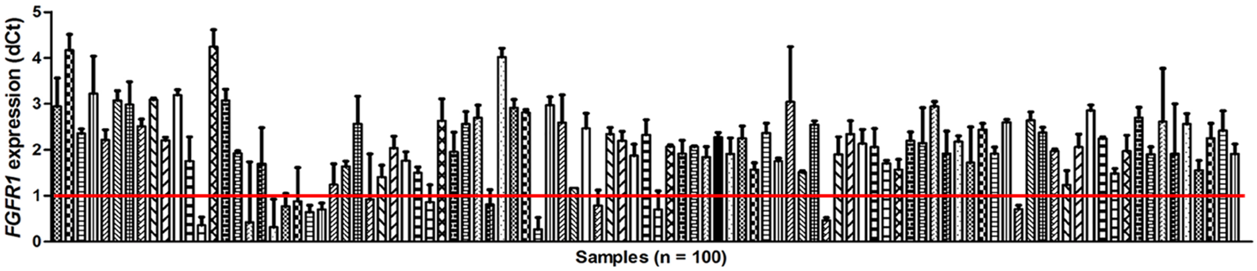

Supplementary Figure 1: Real time PCR validation of *FGFR1* amplification in lung squamous tumors. The bars represent relative dCT of *FGFR1* with respect to *GAPDH* in genomic DNA of lung squamous tumors. The red line indicates dCt equal to 1 and sample with dCt < 1 are considered to harbour FGFR1 amplification.

Supplementary Table 1: Clinical and histopathological characterstics details of 430 lung squamous patients. See Supplementary Table 1

Supplementary Table 2: List of mutations assayed using single base extension mass spectrometry. See Supplementary Table 2

Supplementary Table 3: Exome sequencing data statistics of 20 lung squamous carcinoma samples

Sr No	ADlabID	Total reads	Mapped reads	Mapping (%)	Coverage (X)	Duplication rate (%)	Tumor content (%)
1	AD2320	29005996	28986749	99.93%	47	19.28%	45.68
2	AD2340	31570028	31534614	99.89%	38	16.67%	42.55
3	AD2385	26407140	26374789	99.88%	39	16.48%	48.90
4	AD2315	34310693	34287481	99.93%	53	21.88%	45.82
5	AD2356	30732407	30703381	99.91%	47	19.96%	44.75
6	AD2351	40041484	39998944	99.89%	62	23.79%	46.17
7	AD2341	32358920	32346084	99.96%	49	19.70%	46.77
8	AD2367	28677072	28668165	99.97%	45	18.26%	45.11
9	AD2375	35895257	35882849	99.97%	58	22.42%	49.89
10	AD2349	33199022	33187437	99.97%	52	20.45%	46.11
11	AD2313	27300847	27294842	99.98%	43	18.98%	45.31
12	AD2378	34122958	34114740	99.98%	55	21.74%	47.01
13	AD2327	36452374	36444685	99.98%	59	22.84%	44.48
14	AD2382	33015446	33007791	99.98%	50	21.03%	46.38
15	AD2374	34709383	34693455	99.95%	56	22.23%	46.42
16	AD2352	41299524	41289402	99.98%	65	24.09%	47.21
17	AD2398	29213834	29206025	99.97%	46	19.55%	44.98
18	AD2364	32445900	32438755	99.98%	51	21.38%	43.44
19	AD2373	35759070	35751965	99.98%	59	24.37%	44.39
20	AD2371	37801701	37793796	99.98%	62	24.65%	46.35

Supplementary Table 4: Total number of transitions and transversions per sample after exome sequencing in lung squamous patients. See Supplementary Table 4

Supplementary Table 5: List of variants after whole exome sequencing in 20 lung squamous samples. See Supplementary Table 5

Supplementary Table 6: Genetic alterations obtained from mass array genotyping of 430 lung squamous tumors. See Supplementary Table 6

Supplementary Table 7: Correlation of clinicopathological features and FGFR1 amplification in Indian Lung Squamous patients

Clinicopathological features	Variable	Number (%), along the column	FGFR1 amplification, Number (%) along the row		*P* value
			FGFR1 amplification		
			Amplified	Not amplified	
Age	<65 years	69	9 (13%)	60 (87%)	0.683
	>65 years	30	5 (16%)	25 (84%)	
Gender	Females	15	3 (20%)	12 (80%)	0.48
	Males	84	11 (13%)	73 (87%)	
Tumor stage	II	5	2 (40%)	3 (60%)	0.103
	III	16	4 (25%)	12 (75%)	
	IV	50	5 (10%)	45 (90%)	
	Information not available	18			
Smoking status	Non-smoker	12	2 (17%)	10 (83%)	0.766
	Smoker	67	9 (13.5%)	58 (86.5%)	
	Information not available	20			

*Indicates statistically significant corelation.
